# In Vivo Computed Tomography as a Research Tool to Investigate Asthma and COPD: Where Do We Stand?

**DOI:** 10.1155/2012/972479

**Published:** 2012-01-11

**Authors:** Gaël Dournes, Michel Montaudon, Patrick Berger, François Laurent

**Affiliations:** ^1^Department of Thoracic and Cardiovascular Imaging, CHU Bordeaux, Avenue de Magellan, 33604 Pessac, France; ^2^Laboratory of Cellular Respiratory Physiology, Centre de Recherche Cardio-Thoracique de Bordeaux, INSERM U1045, University Bordeaux Segalen. 146, rue Léo Saignat, 33076 Bordeaux, France

## Abstract

Computed tomography (CT) is a clinical tool widely used to assess and followup asthma and chonic obstructive pulmonary disease (COPD) in humans. Strong efforts have been made the last decade to improve this technique as a quantitative research tool. Using semiautomatic softwares, quantification of airway wall thickness, lumen area, and bronchial wall density are available from large to intermediate conductive airways. Skeletonization of the bronchial tree can be built to assess its three-dimensional geometry. Lung parenchyma density can be analysed as a surrogate of small airway disease and emphysema. Since resident cells involve airway wall and lung parenchyma abnormalities, CT provides an accurate and reliable research tool to assess their role in vivo. This litterature review highlights the most recent advances made to assess asthma and COPD with CT, and also their drawbacks and the place of CT in clarifying the complex physiopathology of both diseases.

## 1. Introduction

Asthma and chronic obstructive pulmonary disease (COPD) are the most common airway diseases worldwide and affect millions of people, with an increasing incidence. Asthma is characterized by a reversible airway obstruction and a bronchial hyperreactivity in response to a stimulus. Conversely COPD is defined as a chronic airway obstruction which is progressive and poorly reversible [1, 2]. Both diseases are associated with environmental factors such as allergens, viruses, bacteria, or toxics leading to an inflammatory response in patients genetically susceptible. Airway inflammation triggers oedema and bronchial wall infiltration by resident cells [3] (neutrophils, macrophages, mast cells [4], and eosinophils in asthma). Chronic inflammation leads to airway remodelling, and despite similarities, many clinical and pathological features show that the two diseases are distinct [5, 6]. The epithelium appears to be more fragile in asthma, and the epithelial membrane thickness and the bronchial smooth muscle are thicker than in COPD. Emphysema does not occur in asthmatic nonsmoker. In COPD, the epithelium displays mucous metaplasia, and inflammation is associated with loss of alveolar attachments, surrounded by peribronchial fibrosis [7]. Destruction of alveolar wall leads to emphysema which is a structural alteration seen in severe COPD. Beyond the immune system, asthma and COPD involve airway and lung parenchyma morphological changes, and computed tomography (CT) appears to be a noninvasive tool to investigate them in vivo [8, 9]. Submillimetric acquisition can be obtained with an isotropic voxel over the whole lung volume, and fully automatic quantification measurements are achievable using commercially available softwares. It provides an accurate research tool, suitable to help understand the complex physiopathology underlying the two diseases, which is still not well known [10]. Clinical, functional, and histological correlations have been reported. This article is focused on the most recent developments made the last decade to improve this technique, their findings and also their limits, and their perspectives. 

## 2. Quantitative Measurement of Airways and Lung Parenchyma Using CT

### 2.1. Quantification of Airway Wall

The rationale of airway wall quantification using CT is the presence of an increased airway wall thickness in asthmatic and COPD patients compared with control subjects [11–15]. The airway intraluminal area (LA) and the total bronchial layer area (WT) are measured, in millimetres square. The wall area (WA) corresponds to the difference WA = WT − LA. WA% represents WA normalised on WT, that is, WA% = (WA/WT) × 100. WA and LA are not independent from body height, so they need to be normalised on body surface area (BSA), to reduce interindividual variation.

A manual method of segmentation has been described first [11]. It consists in tracing a manual region of interest around the internal and external bronchial wall, with a continuous extrapolation. This method is time consuming, exposed to intra- and interobserver variability and parallax error when the reconstructed plane is not strictly perpendicular to the bronchus main axis. Just a few numbers of bronchi divisions are reasonably available using this method, and the trunk of the right apical bronchus is the main target, owing to its geometry, nearly perpendicular to the axial plane.

**Figure 1:**
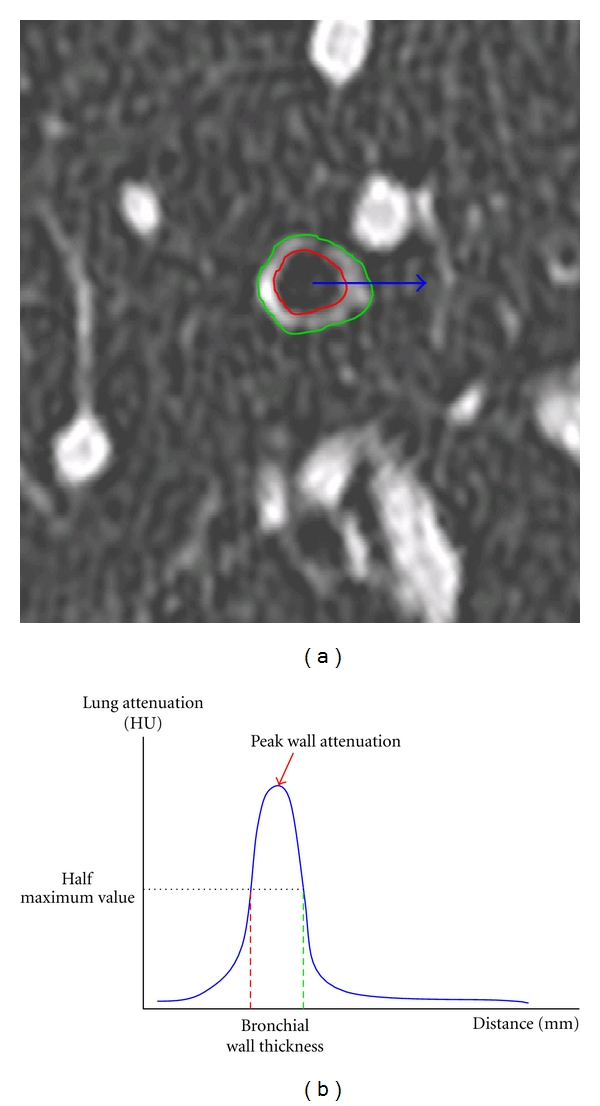
(a) Thin-section CT image perpendicular to the third generation of the right segmental apical bronchus, from a random patient. Red line indicates the external wall contour, and green line the internal layer. (b) Theoric single intensity curve (blue line) representing voxel attenuation variation along the blue arrow seen in right image. The bronchial wall thickness calculated with the FWHM principle is given by the difference between the two extreme values at which the mural portion attenuation is equal to half to its maximum (green and red dashed lines). According to Washko et al., the local Peak Wall Attenuation is given by the maximum attenuation value within the region of interest. Mean bronchial wall thickness and mean Peak Wall Attenuation shall be calculated using a circumferential integration of 128 one-dimensional rays, radiating outward the centroid of the bronchial lumen.

Semiautomatic computational methods have been later developed to allow automated segmentation of the wall contours [12–14] and the bronchial tree [15, 16]. Briefly, active energy-driven contours are a region-based active contour model to extract the local image information. The full-width-at-half-maximum (FWHM) principle is given by the difference between the two extreme values at which the wall attenuation is equal to half to its maximum (Figure 1). The Laplacian-of-Gaussian algorithm is a function that combines a Laplace operator to detect edges as well as noise, and a convolution with a Gaussian kernel to smooth the image first. These algorithms have been used to segment airways wall contours, but none of them have demonstrated any superiority from each other. However, Brillet et al. have proven that they are not interchangeable in longitudinal studies [14].

**Figure 2:**
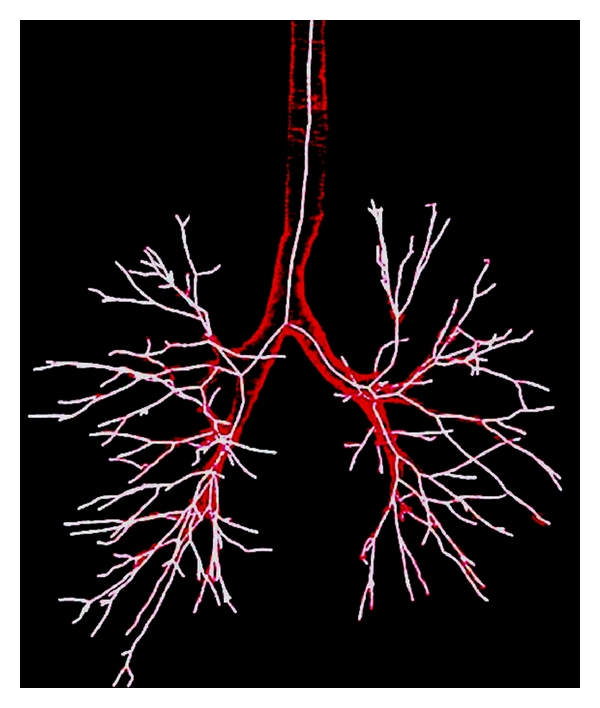
Bronchial tree volume automatically segmented using homemade dedicated software, extracted from a whole set of lung CT images. The skeleton of the bronchial tree is computed to obtain a simplified three-dimensional geometry of the bronchial tree.

Fetita et al. [15] and Montaudon et al. [16] have reported three-dimensional softwares to segment semiautomatically the bronchial tree (Figure 2). Perpendicular planes across the targeted bronchi can be acquired, and WA indices are automatically extracted. These softwares allow a fast and accurate postprocessing quantification, and this is relevant knowing the heterogeneity of alterations in asthma. However, the bronchial human tree displays a mean of 24 divisions including the trachea, and only 10 divisions are reasonably achievable using either manual or semiautomatic methods. Small conductive airways less than 1-2 mm diameter are not clearly visible on CT scans.

Another quantitative parameter has recently been assessed in both asthma and COPD: the bronchial wall attenuation [17–19]. Lederlin et al. [17], in a murine model of asthma, measured the peribronchial attenuation (PBA) using micro-CT with a spatial resolution of 46 microns. The manual method described in their study consisted in a manual segmentation of the peribronchial area, arbitrarily equal to the radius of the target bronchi lumen. In COPD, Washko et al. [18] and Yamashiro et al. [19] studied the peak wall attenuation (PWA) value, extracted from bronchial wall single-intensity curves based on FWHM principle (Figure 1). The mean PWA was calculated by taking the mean peak attenuation along 128 one-dimensional mural rays, radiating outward from the centroid of the airway lumen, using a circumferential measure.

### 2.2. Quantification of Lung Parenchyma

Small conductive and distal airways are beyond the spatial resolution of CT. Intralobular structures are not clearly visible, such as alveolar membranes, capillaries, or interstitial tissue. However, lung parenchyma density is a consequence of the X-ray attenuation by these lung structures, and any change in either of them may modify it. Therefore, lung attenuation provides an indirect tool to assess structural changes in distal airways, though it is nonspecific [20].

Lung alterations can be seen on CT images such as centrilobular micronodules, ground-glass opacities, mosaic pattern, air trapping, and emphysema and have been described in both pathologies [21, 22]. Quantification of these abnormalities has been studied through visual grading, but this method is potentially exposed to variability [23].

In asthma, Mikos et al. [24] measured air trapping on CT scans thanks to a manual method, at a window level of −600 HU and a window width of 1600 HU. Focal air trapping was assessed on end-expiratory scans, superimposing a 10 × 10 mm grid. The number of squares containing low lung attenuation was counted manually in every lung section. Diffuse air trapping was assessed as the ratio between mean lung density in expiration and inspiration (E/I ratio). Landmarks to match inspiratory and expiratory scans were placed at five levels, on superior margin of the aortic arch, tracheal carina, 1 cm below the carina, inferior pulmonary veins, and 2 cm above the diaphragm.

**Figure 3:**
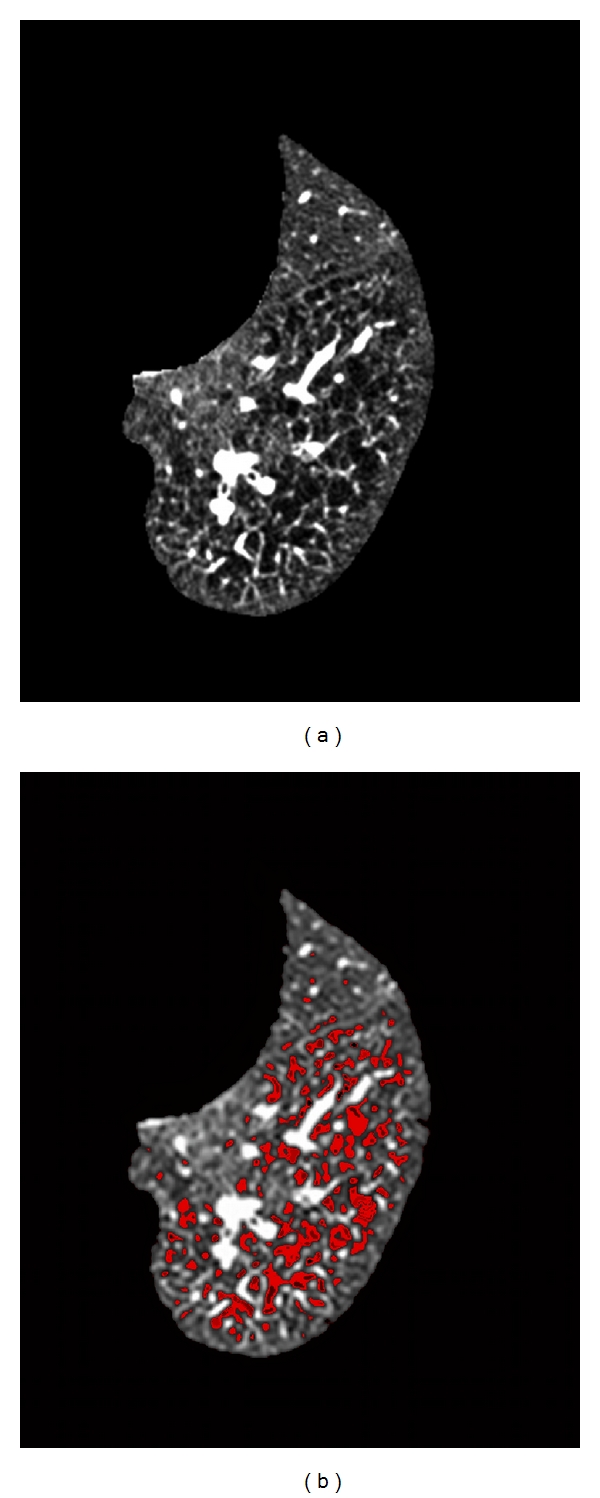
(a) Segmented thin-section CT image of basal left lung area in which lung contours and mediastinum were removed. (b) Same image using the density mask technique.

**Figure 4:**
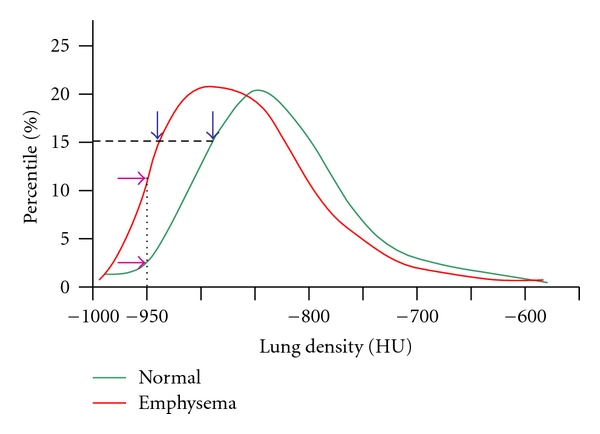
Theoric models of voxel attenuation frequencies in a normal subject (green curve) and emphysematous patient (red curve). The density mask technique is defined as the percentage of total lung volume that contains voxels lower than a predefined voxel index, usually −950 HU to assess emphysema (purple arrows). The percentile method is based on predefined percentages at which voxels have lower attenuation values. Blue arrows indicate the crossing points of green and red curves with the 15th percentile.

Two semiautomatic methods have been further developed [25, 26]. The rationale is the lower lung attenuation measured in emphysema and air trapping areas compared with normal areas. (a) The density mask technique [25] is based on a predefined voxel as a threshold to differentiate between areas of normal attenuation values, and areas of low attenuation (LAA). The density mask technique is defined as the percentage LAA% of total lung volume that contains voxels of lower attenuation values, usually lower than −960 UH in COPD to assess emphysema (Figure 3). (b) The percentile method [26] is based on predefined percentages (1%, 5%, 10%, and 15%) at which voxels have lower attenuation values (Figure 4).

Some drawbacks of these methods have been reported. Since lung attenuation values are not the same between different levels of radiation doses, CT manufacturers [27], or postprocessing softwares [28], Bakker et al. suggested that a calibration of air and blood should be performed before multicenter or longitudinal clinical trials and showed that normalisation of CT quantitative measurements by mean air attenuation value can reduce the variability.

Age and lung volume involve variation of the voxel attenuation values, but not sex gender [29]. Densities are not the same on inspiration or expiration CT scans [30, 31]. The 15th percentile method has been reported to be more independent from lung volume changes than the density mask. Stoel et al. recommend adjusting the 15th percentile to the lung volume to reduce variability in followup studies [32].

Attenuation values are modified when CT is performed with or without contrast injection. Heussel et al. showed higher density in the lung parenchyma after contrast application. Therefore, the amount of emphysema may be underestimated, and they concluded that nonenhanced CT scans should be the reference [33].

## 3. Quantitative CT in Asthma

### 3.1. Large and Intermediate Airway Assessment in Asthma

Asthma involves both proximal and distal airways [1, 2]. Several studies have shown that airway wall thickness (WA) indices are increased in asthmatic patients compared with healthy volunteers [34–37]. According to histological data coming from autopsy studies of fatal cases, this may result from inflammatory changes such as oedema and infiltration of inflammatory cells, and structural changes such as an increased basal membranous thickness, smooth muscle cell layer and peribronchial fibrosis. From bronchial biopsies, Aysola et al. found that WA/LA ratio reflect increase in epithelial, and lamina reticularis thickness [38]. Montaudon et al. found that the slope and the maximal local slope of the WA/LA ratio both correlated with the subepithelial membrane thickness [39]. They showed that the bronchial geometric parameters correlated with smooth muscle area and with infiltration of the smooth muscle by mast cells.

The link between airway thickness measured on CT scans and bronchial reactivity (AHR) is controversial. The most common accepted theory is that part of the airway wall thickness is due to an increased smooth muscle cell layer, which is leading to AHR. Boulet et al. found a positive correlation between airway wall thickness and bronchial hyperreactivity, measured as a fall of 20% of forced expiratory volume in one second (FEV1) after a provocative concentration of metacholine [34]. However, Niimi et al. found that airway sensitivity was related to sputum eosinophil count but not to airway thickness. They also showed a negative correlation between airway thickness and bronchial reactivity, unrelated to eosinophil count. They concluded that airway walls are stiff when thickened, indicating that remodelled asthmatic airways are less distensible and may explain chronic airway obstruction [35]. In other studies, the same authors showed that WA indices are increased in severe as in mild-to-moderate patients with asthma compared with control subjects. In addition, they showed that WA indices correlate with the duration of disease, the severity, and the degree of airflow obstruction [36].

Data around intraluminal area (LA) are controversial too. Niimi et al. [36] and Aysola et al. [38] did not find any significant difference between asthmatic patients and controls. Lynch et al. reported that 77% of asthmatic patients had an internal bronchial diameter to pulmonary artery ratio >1.0, indicating bronchial dilatation [37]. Conversely, Montaudon et al. [39] and Beigelman-Aubry et al. [40] reported a bronchial cross-sectional area significantly smaller in asthmatic than in healthy volunteers. These different features may be explained by heterogeneity of bronchial diameters in asthma. For instance, Niimi et al. measured the right apical segmental bronchus, whereas Aysola et al. quantified the first to the third generation, and Montaudon et al. from the fourth to the tenth.

Peribronchial density has been recently assessed by Lederlin et al. in a murine model of asthma [17]. They did not quantified WA or LA, but micro-CT peribronchial density (PBA), and showed that the attenuation around the bronchial tree in asthmatic mice was increased compared with controls. This increase correlated with both inflammation and remodelling features.

CT bronchial dimensions have been studied to assess medication effects. Kurashima et al. used CT to evaluate the efficacy of inhaled corticosteroid and found a decrease in airway wall thickness among asthmatic patients with duration of symptoms less than 3 years, a minor response among 3 to 5 years and no change in wall thickness in patients with more than 5 years duration of disease [41]. However, Brillet et al. did not found any change of both WA and LA after a combination of salmeterol/flucitasone daily for 12 weeks, though patients displayed clinical and functional improvement, assessed by a decrease in FEV1 and expiratory reserve volume (ERV) [42].

### 3.2. Lung Parenchyma Assessment in Asthma

Asthma is a predominant airway diseases and does not involve lung parenchyma destruction during stable stages [1, 2]. However, CT lung parenchyma changes have been reported.

Using a visual grading, Laurent et al. observed that the mosaic perfusion pattern was significantly increased at full inspiration in 22 patients with stable moderate asthma (23%), compared with 12 healthy nonsmoker [21]. This result was addressed to either hypoxic vasoconstriction or small airway obstruction. They also found that air trapping was increased in asthmatic and healthy smokers, but not in controls. In asthmatic patients, air trapping scores correlated with FEV1 and FEF25–75%, and this was ascribed to small airway obstruction.

Mikos et al. using a manual method, showed that focal and diffuse air trapping (E/I ratio) correlated with airway wall thickness (WA%) [24]. Focal air trapping was significantly increased in a subgroup of 10 asthmatics patients with normal FEV1% predicted and FEV1/FVC%.

**Figure 5:**
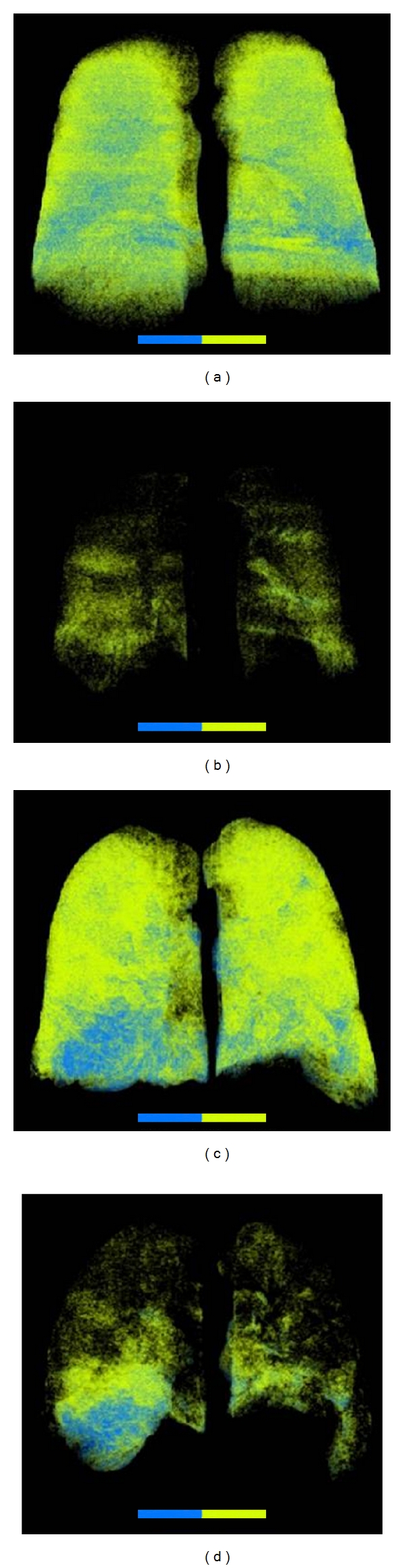
Ventral views of lung attenuation volume samples, computed using the density mask technique to extract voxels below −850 HU. Green areas are representative of voxels between −850 and −900 HU, and blue areas between −900 and −950 HU. Images (a) and (b) were acquired with spirometrically gated CT scans in a non-severe asthmatic subject, in inspiration (a) and in expiration (b). Same images were acquired at same levels of inspiration (c) and expiration (d) from a severe asthmatic subject.

In a multivariate analysis of risk factors, Busacker et al. studied 60 patients with severe asthma, 34 nonsevere asthma and 26 controls. Using a semiautomatic method of CT quantification based on the density mask technique, he defined air trapping as areas of attenuation lower than −850 HU on CT scans acquired in expiration. Air trapping was considered significant whether more than 9.66% of the whole lung volume was involved (Figure 5). They analysed that patients with the air trapping phenotype are more likely to have a history of asthma-related hospitalizations and mechanical ventilation. Several risk factors of this phenotype where noted such as a history of pneumonia, neutrophilic inflammation, and atopy [43].

Mitsunobu et al. evaluated the heterogeneity of asthma disease using LAA% and a fractal analysis, to extract a D coefficient as a surrogate of small airway geometry complexity [44]. They found that LAA% and D correlated in a subset of asthmatic smokers, but not in nonsmokers. LAA% was different in mild and moderate asthma, but D was not. They concluded that D was a biomarker of emphysematous changes, which can help to characterize areas of low attenuation.

Lung density has been used to evaluate CT changes after therapy. Mitsunobu et al. demonstrated that mean lung density (MLD) and relative lung areas of attenuation under −950 HU were improved after systemic glucocorticoid therapy [45]. MLD and LAA% under −950 HU both correlated with FEV1% improvement after therapy.

## 4. Quantitative CT in COPD

### 4.1. Large and Intermediate Airway Assessment in COPD

Small airways are the main site of obstruction in COPD [1, 2]. However, large airways are not free of abnormalities. Lee et al. defined a tracheal index (TI) as the ratio between the tracheal diameter measured on the coronal plane, and the sagittal diameter. They showed significant correlation between TI and severity of emphysema [46]. Sverzelatti et al. studied the prevalence of bronchial diverticula in smokers [47]. Grade 2 was defined as the presence of more than three diverticulas in large airways. This feature correlated with a more frequent history of cough, a greater extent of emphysema, a more severe bronchial wall thickening, and a heavier level of smoking.

Nakano et al. demonstrated that the mean dimensions of large and intermediate airways with an internal perimeter greater than 0.75 cm predicted the mean dimensions of small airways with an internal diameter of 1.25 mm [48].

Several studies have shown that airway wall thickness correlates with pulmonary function tests (PFTs) [49–53]. In a study conducted in 114 smokers, Nakano et al. showed that WA% measured on the trunk of the right apical bronchus correlated with FEV1 predicted, forced vital capacity (FRC), and residual volume/total lung capacity (RV/TLC) [49]. Grydeland et al. demonstrated that DLCO correlates with both emphysema and airway wall thickness [50], though Nakano et al. did not find significant correlation. Berger et al. measured airway dimensions with spirometrically gated CT. They showed that normalized WA and LA correlated with FEV1 and FEF25%–75% in smokers with and without COPD. Moreover, these dimensions were significantly larger in smokers with COPD than in smokers without COPD or non smokers [51].

Achenbach et al. wondered whether the strong correlations calculated between small airways dimensions and PFT is overestimated by the point spread function (PSF) artefact, or not. PSF involves blurring of the small airways contours and can lead to overestimate them using the FWHM algorithm. Using another three-dimensional approach taking into account the PSF, they assessed a median of 619 orthogonal airway locations per patient. They observed a significant correlation between airway dimensions and FEV1 in COPD patients. This correlation was higher from large to small airways, which is in agreement with FWHM principle [54].

Shimizu et al. have compared airway dimensions in 28 male COPD versus 12 sex and age-matched asthmatic and 13 age-matched healthy smokers. WA% and LA were measured from the 3rd to the 6th generation. At any generation, WA% was smaller and LA larger in COPD than in asthma, followed by controls. FEV1 predicted and FEV1/FVC was similar between asthma and COPD. They concluded that remodelling is more prominent in asthma than in COPD under stable clinical conditions [55].

Bronchial wall attenuation has been recently assessed in COPD. Washko et al. and Yamashiro et al. used the FWHM algorithm to extract the Peak Wall Attenuation value from the bronchial wall, as a surrogate of its main density. They showed strong correlations between this new biomarker of airway wall structural changes and airway obstructions assessed by PFT. Correlations were stronger in small airways. However, PWA extracted with FWHM principle is not independent from airway wall dimension [18, 19]. 

### 4.2. Lung Parenchyma Assessment in COPD

Ex vivo studies in isolated lungs and in vivo invasive measurements of airway resistance revealed that distal airways are the main site of airflow obstruction in COPD [5, 6]. Pathological studies highlighted that the small conductive airways are infiltrated by phagocytes (macrophages and neutrophils), dendritic cells, and T and B lymphocytes. Structural changes include airway wall thickness and obstruction by muco-inflammatory exudates and emphysema. Lung density provides an indirect tool to assess them in vivo, though non specific.

Centrilobular nodules and branching lines are areas of high attenuation and reflect pathological changes in small conductive airways, either inflammation or fibrosis. Destruction of alveolar walls is the hallmark of emphysema, and this pathological feature induces decreased areas of lung attenuation. Using the density mask and the percentile methods, Madani et al. showed that the −960 HU voxel index and the 1st percentile correlate with emphysema extent on pathological examinations [56]. Gevenois et al. showed that expiratory quantitative CT is not as accurate as inspiratory CT to measure lung emphysema [57].

However, areas of decreased attenuation can be visible on inspiratory images as a mosaic pattern, and air trapping on expiratory images. Both obstruction of the small conductive airways and loss of alveolar attachments are associated with destabilisation and premature airway closure during expiration. Therefore, differentiating emphysema from air trapping is not reliably achievable on CT images when assessed visually. Nevertheless, Matsuoka et al. have developed a quantitative method to evaluate it. They suggested that voxels <950 HU represent emphysema, and the relative volume changes, between inspiration and expiration, of voxels between −950 HU and −860 HU is thought to reflect air trapping [58–61].

Another paradoxical fall in lung density has been reported by Shaker et al. in COPD smokers. LAA% with a voxel index of −910 HU displayed a rapid fall in lung density after smoking cessation, mimicking rapid progression of emphysema. This was ascribed to an anti-inflammatory effect of smoking cessation and is not to be misinterpreted [62]. Persistent airway inflammation and emphysema progression have been showed in exsmokers after 4-year smoking cessation [63].

Correlations between the extent of emphysema and pulmonary function tests have been long reported [64–69]. COPD is characterized by expiratory airflow limitation that results in delayed emptying of the lung, poorly reversible. LAA% has been shown to correlate with FVC% predicted, FEV1% predicted, FEV1/FVC, RV/TLC, and DLCO/VA. Gurney et al. studied emphysema distribution and showed that predominantly lower lobe zones of emphysema are more likely to correlate with obstructive dysfunction and DLCO [70].

CT quantification has been assessed as a predictor of lung function decline in smokers with normal PFTs. Tsushima et al. reported that abnormal CT findings were predictive of airflow limitation and development of emphysema in smokers with normal FEV1 [71]. Yuan et al. demonstrated that CT quantification of overinflation is predictive of FEV1 decline in smokers with normal lung function [72]. Using the 15th percentile, Hoesein et al. demonstrated that the extent of emphysema quantified by CT correlates with the lung function decline assessed by FEV1 at 3-year followup [73].

CT has been used to explore clinical outcomes associated with COPD. Mair et al. [74], and Ogawa et al. [75] reported a negative correlation between LAA% and body mass index (BMI), and emphysema dominant COPD phenotype showed stronger negative correlation with BMI than airway dominant. Ohara et al. reported that LAA% correlates with significant reduced bone density, as a biomarker of osteoporosis in COPD patients [76]. Independent associations with thoracic calcification have been reported by Dransfield et al., suggesting that LAA% can be used as a risk factor of cardiovascular disease in patients with and without COPD [77]. Emphysema is associated with an increased risk of lung cancer [78, 79]. Gullón et al. showed that presence of emphysema in patients with nonsmall cells lung cancer affect the survival rate and can be consider a prognostic factor [80]. Haruna et al. have followed up 251 COPD patients. Among them, 79 died, and 40 deaths were attributable to respiratory disease not involving lung cancer. A multivariate analysis comparing age, PFT, BMI, and emphysema assessed by CT revealed that LAA% had the strongest association with mortality [81].

Using CT quantification, Shaker et al. did not find significant correlation between CT emphysema quantification and emphysema progression after corticosteroid therapy [82]. Nevertheless, lung attenuation has been reported to be a predictor of outcome after lung volume reduction surgery in severe COPD. Wahsko et al. showed weak but statistically significant correlation between emphysema CT measures and 6-month postoperative outcomes assessed by FEV1 and maximal exercise changes [83]. Sciurba et al. demonstrated a poorer survival postoperative rate in patients with increased LAA%. Both studies did not show any significant correlation with airway wall thickness [84].

### 4.3. COPD Classification Using CT

According to the Global Initiative for Chronic Obstructive Lung Disease (GOLD) guideline, COPD is a disease state characterized by airflow limitation that is not fully reversible. Irreversible airflow limitation is defined as FEV1/FVC < 70% after inhalation of *β*2-agonist. Chronic bronchitis and emphysema are not included in the GOLD definition, because they do not allow accurate discrimination based on clinical symptomatic features [85].

Nakano et al. suggested that CT is useful to discriminate between patients who have primarily parenchyma disease from those who have primarily airway pathology. Using WA% and LAA% as objective and quantitative surrogates of, respectively, airway and lung disease, they found that they could divide COPD patients into groups; airway remodelling-dominant group (high WA% and low LAA%), emphysema dominant group (low WA% and high LAA%), and a mixed group (high WA% and high LAA%). They did not study the clinical features associated with this classification based on morphological CT changes [86].

Fujimoto et al. classified COPD patients into three morphological groups and studied their clinical and functional signification. The A phenotype was defined as absence of emphysema with or without bronchial wall thickening, E phenotype as emphysema without wall thickening, and M phenotype as a combination of emphysema and bronchial wall thickening. They showed that the clinical features between these three phenotypes were different. The A phenotype showed a higher prevalence of nonsmoker COPD patients, higher BMI and DLCO, and milder hyperinflation compared with the E. The M phenotype showed a higher prevalence of severe COPD, assessed by sputum level, productive cough, wheezing, exacerbation, and hospitalizations. A and M phenotypes showed greater airflow reversibility of airflow limitation responsive to *β*2-agonist inhalation compared with E [87, 88].

Fujimoto et al. studied the efficacy of long-acting muscarinic antagonist (tiotropium) in COPD patients, classified in dominant emphysema or nondominant on their morphological CT scans. Tiotropium improved airflow limitation in all types, regardless to the emphysema dominance, and dynamic hyperinflation in the emphysema dominant phenotype [89].

## 5. Perspectives

Strong efforts have been made the last decade to assess CT as a research tool to understand the role of resident cells in asthma and COPD. This literature data screening show that clinical and animal study is now available in vivo thanks to MDCT technology. However, a cellular level of detection has not been reached yet. Further developments need to be performed to obtain cellular-specific quantifications.

Spatial resolution should be improved. Small conductive airways inferior to 1 mm are not clearly visible on CT images, and they still need to be addressed. An indirect CT parameter is provided by lung attenuation. Lung attenuation is though nonspecific and may be altered by any change in intralobular structures. Blurring effect may also modify CT wall thickness quantification of small to intermediate airways. A better delineation of these structures may allow a better understanding of their alterations in asthma and COPD.

Wall density is a new biomarker in asthma and COPD which need further development. Only nonenhanced wall attenuation has been reported. No data exists about CT-enhanced wall attenuation value changes. For instance, fibrosis is typically characterized in organs by a delayed enhancement on CT scans using an iodine contrast medium, and data around peribronchial fibrosis in COPD or chronic asthma have not been reported. Specific contrast medium should be developed to enhance target cells, and density may be the one tool to quantify possible enhancement.

Postprocessing softwares are to be improved. Just a few numbers of bronchial generations are available for 3D segmentation. The gradient of density between bronchial and parenchyma air attenuation is not suitable for an accurate segmentation beyond a few number of bronchi generations.

MRI techniques using noble hyperpolarized gases have been reported, but they are cost effective and not suitable for a routine clinical or research practice widely used [90–92]. To our knowledge, no data exists about proton MRI of bronchi wall quantification. Enhanced MRI using gadolinium medium or others in bronchi wall is not documented. Due to its inocuity, MRI should provide cine images of bronchi diameter modifications under physiologic or pathologic conditions.

## 6. Conclusion

CT is an accurate tool to investigate in vivo asthma and COPD physiopathology. Quantification of airway wall and lung parenchyma has demonstrated strong correlations with clinical, functional, and pathological features, in humans or in animal models. However, spatial resolution and specific contrast medium need to be further developed to allow a cellular level of detection which has not been reached yet.

## Abbreviations

COPD: Chronic obstructive pulmonary disease
CT: Computed tomography
LA: Luminal area
WT: Wall layer area
WA: Wall area
BSA: Body surface area
MLD: Mean lung density
FWHM: Full-width-at-half-maximum
PWA: Peak wall attenuation
HU: Hounsfield Unit
LAA: Lung attenuation area
AHR: Airway hyper reactivity
PFT: Pulmonary function testing
FEV1: Forced expiratory volume in 1 second
ERV: Expiratory residual volume
FEF25%–75%: Forced expiratory flow 25%–75%
FVC: Forced vital capacity
FRC: Forced residual capacity
RV: Residual volume
TLC: Total lung capacity
DLCO: Diffusing capacity of the lung for carbon monoxide
TI: Tracheal index
PSF: Point spread function
BMI: Body mass index
MDCT: Multidetector computed tomography
MRI: Magnetic resonance imaging.

## References

[1] Sciurba FC (2004). Physiologic similarities and differences between COPD and asthma. *Chest*.

[2] Barnes PJ (2006). Against the Dutch hypothesis: asthma and chronic obstructive pulmonary disease are distinct diseases. *American Journal of Respiratory and Critical Care Medicine*.

[3] Barnes PJ (2008). Immunology of asthma and chronic obstructive pulmonary disease. *Nature Reviews Immunology*.

[4] Tunon-de-Lara JM, Berger P, Bégueret H, Brightling CE, Bradding P, Pavord ID (2002). Mast cells in airway smooth muscle. *The New England Journal of Medicine*.

[5] Hogg J (2004). Peripheral lung remodelling in asthma and chronic obstructive pulmonary disease. *European Respiratory Journal*.

[6] Hogg JC, McDonough JE, Gosselink JV, Hayashi S (2009). What drives the peripheral lung-remodeling process in chronic obstructive pulmonary disease?. *Proceedings of the American Thoracic Society*.

[7] Burgel PR, de Blic J, Chanez P (2009). Update on the roles of distal airways in asthma. *European Respiratory Review*.

[8] Niimi A, Matsumoto H, Takemura M, Ueda T, Nakano Y, Mishima M (2004). Clinical assessment of airway remodeling in asthma: utility of computed tomography. *Clinical Reviews in Allergy and Immunology*.

[9] Nakano Y, Van Tho N, Yamada H, Osawa M, Nagao T (2009). Radiological approach to asthma and COPD-the role of computed tomography. *Allergology International*.

[10] Aubier M, Marthan R, Berger P (2010). COPD and inflammation: statement from a French expert group: inflammation and remodelling mechanisms. *Revue des Maladies Respiratoires*.

[11] Nakano Y, Muro S, Sakai H (2000). Computed tomographic measurements of airway dimensions and emphysema in smokers correlation with lung function. *American Journal of Respiratory and Critical Care Medicine*.

[12] Nishimura M (2008). Application of three-dimensional airway algorithms in a clinical study. *Proceedings of the American Thoracic Society*.

[13] San José Estépar R, Reilly JJ, Silverman EK, Washko GR (2008). Three-dimensional airway measurements and algorithms. *Proceedings of the American Thoracic Society*.

[14] Brillet PY, Fetita CI, Capderou A (2009). Variability of bronchial measurements obtained by sequential CT using two computer-based methods. *European Radiology*.

[15] Fetita CI, Prêteux F, Beigelman-Aubry C, Grenier P (2004). Pulmonary airways: 3-D reconstruction from multislice CT and clinical investigation. *IEEE Transactions on Medical Imaging*.

[16] Montaudon M, Berger P, Lederlin M, Marthan R, Tunon-de-Lara JM, Laurent F (2009). Bronchial morphometry in smokers: comparison with healthy subjects by using 3D CT. *European Radiology*.

[17] Lederlin M, Ozier A, Montaudon M (2010). Airway remodeling in a mouse asthma model assessed by in-vivo respiratory-gated micro-computed tomography. *European Radiology*.

[18] Washko GR, Dransfield MT, Estépar RSJ (2009). Airway wall attenuation: a biomarker of airway disease in subjects with COPD. *Journal of Applied Physiology*.

[19] Yamashiro T, Matsuoka S, San José Estépar R (2010). Quantitative assessment of bronchial wall attenuation with thin-section CT: an indicator of airflow limitation in chronic obstructive pulmonary disease. *American Journal of Roentgenology*.

[20] Gevenois PA, Yernault JC (1995). Can computed tomography quantify pulmonary emphysema?. *European Respiratory Journal*.

[21] Laurent F, Latrabe V, Raherison C, Marthan R, Tunon-De-Lara JM (2000). Functional significance of air trapping detected in moderate asthma. *European Radiology*.

[22] Gupta S, Siddiqui S, Haldar P (2009). Qualitative analysis of high-resolution CT scans in severe asthma. *Chest*.

[23] Ledenius K, Svensson E, Stålhammar F, Wiklund LM, Thilander-Klang A (2010). A method to analyse observer disagreement in visual grading studies: example of assessed image quality in paediatric cerebral multidetector CT images. *British Journal of Radiology*.

[24] Mikos M, Grzanka P, Sladek K (2009). High-resolution computed tomography evaluation of peripheral airways in asthma patients: comparison of focal and diffuse air trapping. *Respiration*.

[25] Gevenois PA, de Maertelaer V, de Vuyst P, Zanen J, Yernault JC (1995). Comparison of computed density and macroscopic morphometry in pulmonary emphysema. *American Journal of Respiratory and Critical Care Medicine*.

[26] Madani A, Zanen J, de Maertelaer V, Gevenois PA (2006). Pulmonary emphysema: objective quantification at multi-detector row CT - Comparison with macroscopic and microscopic morphometry. *Radiology*.

[27] Yuan R, Mayo JR, Hogg JC (2007). The effects of radiation dose and CT manufacturer on measurements of lung densitometry. *Chest*.

[28] Bakker ME, Stolk J, Putter H (2005). Variability in densitometric assessment of pulmonary emphysema with computed tomography. *Investigative Radiology*.

[29] Gevenois PA, Scillia P, de Maertelaer V, Michils A, de Vuyst P, Yernault JC (1996). The effects of age, sex, lung size, and hyperinflation on CT lung densitometry. *American Journal of Roentgenology*.

[30] Madani A, Van Muylem A, Gevenois PA (2010). Pulmonary emphysema: effect of lung volume on objective quantification at thin-section CT. *Radiology*.

[31] Akira M, Toyokawa K, Inoue Y, Arai T (2009). Quantitative CT in chronic obstructive pulmonary disease: inspiratory and expiratory assessment. *American Journal of Roentgenology*.

[32] Stoel BC, Putter H, Bakker ME (2008). Volume correction in computed tomography densitometry for follow-up studies on pulmonary emphysema. *Proceedings of the American Thoracic Society*.

[33] Heussel CP, Kappes J, Hantusch R (2010). Contrast enhanced CT-scans are not comparable to non-enhanced scans in emphysema quantification. *European Journal of Radiology*.

[34] Boulet LP, Lemière C, Archambault F, Carrier G, Descary MC, Deschesnes F (2006). Smoking and asthma: clinical and radiologic features, lung function, and airway inflammation. *Chest*.

[35] Niimi A, Matsumoto H, Takemura M, Ueda T, Chin K, Mishima M (2003). Relationship of airway wall thickness to airway sensitivity and airway reactivity in asthma. *American Journal of Respiratory and Critical Care Medicine*.

[36] Niimi A, Matsumoto H, Amitani R (2000). Airway wall thickness in asthma assessed by computed tomography: relation to clinical indices. *American Journal of Respiratory and Critical Care Medicine*.

[37] Lynch DA, Newell JD, Tschomper BA, Cink TM, Newman LS, Bethel R (1993). Uncomplicated asthma in adults: comparison of CT appearance of the lungs in asthmatic and healthy subjects. *Radiology*.

[38] Aysola RS, Hoffman EA, Gierada D (2008). Airway remodeling measured by multidetector CT is increased in severe asthma and correlates with pathology. *Chest*.

[39] Montaudon M, Lederlin M, Reich S (2009). Bronchial measurements in patients with asthma: comparison of quantitative thin-section CT findings with those in healthy subjects and correlation with pathologic findings. *Radiology*.

[40] Beigelman-Aubry C, Capderou A, Grenier PA (2002). Mild intermittent asthma: CT assessment of bronchial cross-sectional area and lung attenuation at controlled lung volume. *Radiology*.

[41] Kurashima K, Kanauchi T, Hoshi T (2008). Effect of early versus late intervention with inhaled corticosteroids on airway wall thickness in patients with asthma. *Respirology*.

[42] Brillet PY, Attali V, Nachbaur G (2010). Multidetector Row Computed Tomography to Assess Changes in Airways Linked to Asthma Control. *Respiration*.

[43] Busacker A, Newell JD, Keefe T (2009). A multivariate analysis of risk factors for the air-trapping asthmatic phenotype as measured by quantitative CT analysis. *Chest*.

[44] Mitsunobu F, Ashida K, Hosaki Y (2003). Complexity of terminal airspace geometry assessed by computed tomography in asthma. *American Journal of Respiratory and Critical Care Medicine*.

[45] Mitsunobu F, Mifune T, Ashida K (2001). Low-attenuation areas of the lungs on high-resolution computed tomography in asthma. *Journal of Asthma*.

[46] Lee HJ, Seo JB, Chae EJ Tracheal morphology and collapse in COPD: correlation with CT indices and pulmonary function test.

[47] Sverzellati N, Ingegnoli A, Calabrò E (2010). Bronchial diverticula in smokers on thin-section CT. *European Radiology*.

[48] Nakano Y, Wong JC, de Jong PA (2005). The prediction of small airway dimensions using computed tomography. *American Journal of Respiratory and Critical Care Medicine*.

[49] Nakano Y, Muro S, Sakai H (2000). Computed tomographic measurements of airway dimensions and emphysema in smokers correlation with lung function. *American Journal of Respiratory and Critical Care Medicine*.

[50] Grydeland TB, Thorsen E, Dirksen A (2010). Quantitative CT measures of emphysema and airway wall thickness are related to D_L_CO. *Respiratory Medicine*.

[51] Berger P, Perot V, Desbarats P, Tunon-De-Lara JM, Marthan R, Laurent F (2005). Airway wall thickness in cigarette smokers: quantitative thin-section CT assessment. *Radiology*.

[52] Nieber M, Putter H, Stolk J (2006). Prediction of pulmonary function in COPD on the basis of CT measurements of bronchial wall thickness [2] (multiple letters). *Radiology*.

[53] Kurashima K, Takayanagi N, Sato N (2005). High resolution CT and bronchial reversibility test for diagnosing COPD. *Respirology*.

[54] Achenbach T, Weinheimer O, Biedermann A (2008). MDCT assessment of airway wall thickness in COPD patients using a new method: correlations with pulmonary function tests. *European Radiology*.

[55] Shimizu K, Hasegawa M, Makita H, Nasuhara Y, Konno S, Nishimura M (2011). Comparison of airway remodelling assessed by computed tomography in asthma and COPD. *Respiratory Medicine*.

[56] Madani A, Van Muylem A, de Maertelaer V, Zanen J, Gevenois PA (2008). Pulmonary emphysema: size distribution of emphysematous spaces on multidetector CT images - Comparison with macroscopic and microscopic morphometry. *Radiology*.

[57] Gevenois PA, de Vuyst P, Sy M (1996). Pulmonary emphysema: quantitative CT during expiration. *Radiology*.

[58] Matsuoka S, Yamashiro T, Washko GR, Kurihara Y, Nakajima Y, Hatabu H (2010). Quantitative ct assessment of chronic obstructive pulmonary disease. *Radiographics*.

[59] Matsuoka S, Kurihara Y, Yagihashi K, Hoshino M, Nakajima Y (2008). Airway dimensions at inspiratory and expiratory multisection CT in chronic obstructive pulmonary disease: correlation with airflow limitation. *Radiology*.

[60] Matsuoka S, Kurihara Y, Yagihashi K, Hoshino M, Watanabe N, Nakajima Y (2008). Quantitative assessment of air trapping in chronic obstructive pulmonary disease using inspiratory and expiratory volumetric MDCT. *American Journal of Roentgenology*.

[61] Matsuoka S, Kurihara Y, Yagihashi K, Nakajima Y (2007). Quantitative assessment of peripheral airway obstruction on paired expiratory/inspiratory thin-section computed tomography in chronic obstructive pulmonary disease with emphysema. *Journal of Computer Assisted Tomography*.

[62] Shaker SB, Stavngaard T, Laursen LC, Stoel BC, Dirksen A (2011). Rapid fall in lung density following smoking cessation in COPD. *COPD: Journal of Chronic Obstructive Pulmonary Disease*.

[63] Miller M, Cho JY, Pham A, Friedman PJ, Ramsdell J, Broide DH (2011). Persistent airway inflammation and emphysema progression on CT scan in ex-smokers observed for 4 years. *Chest*.

[64] Heussel CP, Herth FJF, Kappes J (2009). Fully automatic quantitative assessment of emphysema in computed tomography: comparison with pulmonary function testing and normal values. *European Radiology*.

[65] Kim WJ, Silverman EK, Hoffman E (2009). CT metrics of airway disease and emphysema in severe COPD. *Chest*.

[66] Marsh S, Aldington S, Williams MV (2007). Utility of lung density measurements in the diagnosis of emphysema. *Respiratory Medicine*.

[67] Yamashiro T, Matsuoka S, Bartholmai BJ (2010). Collapsibility of lung volume by paired inspiratory and expiratory CT scans: correlations with lung function and mean lung density. *Academic Radiology*.

[68] Pauls S, Gulkin D, Feuerlein S (2010). Assessment of COPD severity by computed tomography: correlation with lung functional testing. *Clinical Imaging*.

[69] Omori H, Nakashima R, Otsuka N (2006). Emphysema detected by lung cancer screening with low-dose spiral CT: prevalence, and correlation with smoking habits and pulmonary function in Japanese male subjects. *Respirology*.

[70] Gurney JW, Jones KK, Robbins RA (1992). Regional distribution of emphysema: correlation of high-resolution CT with pulmonary function tests in unselected smokers. *Radiology*.

[71] Tsushima K, Sone S, Fujimoto K (2010). Identification of occult parechymal disease such as emphysema or airway disease using screening computed tomography. *COPD: Journal of Chronic Obstructive Pulmonary Disease*.

[72] Yuan R, Hogg JC, Paré PD (2009). Prediction of the rate of decline in FEV1 in smokers using quantitative computed tomography. *Thorax*.

[73] Mohamed Hoesein FAA, de Hoop B, Zanen P (2011). CT-quantified emphysema in male heavy smokers: association with lung function decline. *Thorax*.

[74] Mair G, Miller JJ, McAllister D (2009). Computed tomographic emphysema distribution: relationship to clinical features in a cohort of smokers. *European Respiratory Journal*.

[75] Ogawa E, Nakano Y, Ohara T (2009). Body mass index in male patients with COPD: correlation with low attenuation areas on CT. *Thorax*.

[76] Ohara T, Hirai T, Muro S (2008). Relationship between pulmonary emphysema and osteoporosis assessed by CT in patients with COPD. *Chest*.

[77] Dransfield MT, Huang F, Nath H, Singh SP, Bailey WC, Washko GR (2010). CT emphysema predicts thoracic aortic calcification in smokers with and without COPD. *COPD: Journal of Chronic Obstructive Pulmonary Disease*.

[78] Wilson DO, Weissfeld JL, Balkan A (2008). Association of radiographic emphysema and airflow obstruction with lung cancer. *American Journal of Respiratory and Critical Care Medicine*.

[79] Nakajima T, Sekine Y, Yamada V (2009). Long-term surgical outcome in patients with lung cancer and coexisting severe COPD. *Thoracic and Cardiovascular Surgeon*.

[80] Gullón JA, Suárez I, Medina A, Rubinos G, Fernández R, González I (2011). Role of emphysema and airway obstruction in prognosis of lung cancer. *Lung Cancer*.

[81] Haruna A, Muro S, Nakano Y (2010). CT scan findings of emphysema predict mortality in COPD. *Chest*.

[82] Shaker SB, Dirksen A, Ulrik CS (2009). The effect of inhaled corticosteroids on the development of emphysema in smokers assessed by annual computed tomography. *COPD: Journal of Chronic Obstructive Pulmonary Disease*.

[83] Washko GR, Martinez FJ, Hoffman EA (2010). Physiological and computed tomographic predictors of outcome from lung volume reduction surgery. *American Journal of Respiratory and Critical Care Medicine*.

[84] Sciurba FC, Martinez FJ, Rogers RM (2006). Relationship between pathologic characteristics of peripheral airways and outcome after lung volume reduction surgery in severe chronic obstructive pulmonary disease. *Proceedings of the American Thoracic Society*.

[85] Pescarolo M, Sverzellati N, Verduri A (2008). How much do GOLD stages reflect CT abnormalities in COPD patients?. *Radiologia Medica*.

[86] Nakano Y, Müller NL, King GG (2002). Quantitative assessment of airway remodeling using high-resolution CT. *Chest*.

[87] Fujimoto K, Kitaguchi Y, Kubo K, Honda T (2006). Clinical analysis of chronic obstructive pulmonary disease phenotypes classified using high-resolution computed tomography. *Respirology*.

[88] Fujimoto K, Yoshiike F, Yasuo M (2007). Effects of bronchodilators on dynamic hyperinflation following hyperventilation in patients with COPD. *Respirology*.

[89] Fujimoto K, Kitaguchi Y, Kanda S, Urushihata K, Hanaoka M, Kubo K (2011). Comparison of efficacy of long-acting bronchodilators in emphysema dominant and emphysema nondominant chronic obstructive pulmonary disease. *International Journal of Chronic Obstructive Pulmonary Disease*.

[90] Peterson ET, Dai J, Holmes JH, Fain SB (2011). Measurement of lung airways in three dimensions using hyperpolarized helium-3 MRI. *Physics in Medicine and Biology*.

[91] Aysola R, de Lange EE, Castro M, Altes TA (2010). Demonstration of the heterogeneous distribution of asthma in the lungs using CT and hyperpolarized helium-3 MRI. *Journal of Magnetic Resonance Imaging*.

[92] Kirby M, Mathew L, Wheatley A, Santyr GE, McCormack DG, Parraga G (2010). Chronic obstructive pulmonary disease: longitudinal hyperpolarized 3He MR imaging. *Radiology*.

